# Google Trends as a Predictive Tool for COVID-19 Vaccinations in Italy: Retrospective Infodemiological Analysis

**DOI:** 10.2196/35356

**Published:** 2022-04-19

**Authors:** Alessandro Rovetta

**Affiliations:** 1 R&C Research Bovezzo Italy

**Keywords:** COVID-19, epidemiology, Google Trends, infodemiology, infoveillance, Italy, public health, SARS-CoV-2, vaccinations, vaccines, social media analysis, social media

## Abstract

**Background:**

Google Trends is an infoveillance tool widely used by the scientific community to investigate different user behaviors related to COVID-19. However, several limitations regarding its adoption are reported in the literature.

**Objective:**

This paper aims to provide an effective and efficient approach to investigating vaccine adherence against COVID-19 via Google Trends.

**Methods:**

Through the cross-correlational analysis of well-targeted hypotheses, we investigate the predictive capacity of web searches related to COVID-19 toward vaccinations in Italy from November 2020 to November 2021. The keyword “vaccine reservation” query (VRQ) was chosen as it reflects a real intention of being vaccinated (V). Furthermore, the impact of the second most read Italian newspaper (vaccine-related headlines [VRH]) on vaccine-related web searches was investigated to evaluate the role of the mass media as a confounding factor. Fisher r-to-z transformation (*z*) and percentage difference (δ) were used to compare Spearman coefficients. A regression model *V=f(VRH, VRQ)* was built to validate the results found. The Holm-Bonferroni correction was adopted (P*). SEs are reported.

**Results:**

Simple and generic keywords are more likely to identify the actual web interest in COVID-19 vaccines than specific and elaborated keywords. Cross-correlations between VRQ and V were very strong and significant (min *r*²=0.460, P*<.001, lag 0 weeks; max *r*²=0.903, P*<.001, lag 6 weeks). The remaining cross-correlations have been markedly lower (δ>55.8%; *z*>5.8; P*<.001). The regression model confirmed the greater significance of VRQ versus VRH (P*<.001 vs *P*=.03, P*=.29).

**Conclusions:**

This research provides preliminary evidence in favor of using Google Trends as a surveillance and prediction tool for vaccine adherence against COVID-19 in Italy. Further research is needed to establish the appropriate use and limits of Google Trends for vaccination tracking. However, these findings prove that the search for suitable keywords is a fundamental step to reduce confounding factors. Additionally, targeting hypotheses helps diminish the likelihood of spurious correlations. It is recommended that Google Trends be leveraged as a complementary infoveillance tool by government agencies to monitor and predict vaccine adherence in this and future crises by following the methods proposed in this paper.

## Introduction

Google Trends is an online website created by Google LLC that allows the user to examine the popularity of exact search queries (keywords) in Google Search across specific regions, time lapses, and languages. Google Trends has often been used by the scientific community to conduct infodemiological and epidemiological analyses [1,2]. In particular, this infoveillance approach—aimed at studying distribution and determinants of information in an electronic medium, specifically the internet, or in a population, with the ultimate aim to inform public health and public policy—has been applied to various disciplines, including but not limited to psychology, economics, veterinary medicine, and pharmacy [3-7]. However, past studies have been often criticized for not providing sufficient documentation to guarantee the full reproducibility of the methods [8]. Moreover, some authors have shown severe limitations in its use as a surveillance tool, including anomalies in results and mass media influence [9,10]. Nonetheless, Google Trends remains a currently irreplaceable tool for infoveillance. In particular, its simplicity and efficiency make analyses much faster than other systems, such as investigating user posts via application programming interface and machine learning [10]. In this regard, various strategies have been proposed in the literature to address its weaknesses [10-12]. Taking the latter into account, in this brief paper, Google Trends is used to investigate vaccine adherence in Italy against COVID-19. Indeed, COVID-19 vaccines are essential to contain the infection, limiting the spread of new variants of concern and substantially reducing the severity of the disease [13]. For instance, the latest report from the Italian Medicines Agency highlighted a low risk associated with vaccines despite high protection against COVID-19 [14]. Even considering Omicron’s more elusive variant of concern, the rates of hospitalizations, patients in intensive care units, and deaths are 10, 27, and 25 times higher for the unvaccinated, respectively [15]. At present, monitoring of vaccine adherence is epidemiologically essential, especially considering the growing no-vax movement [16]. Furthermore, the use of effective and efficient infoveillance techniques is also necessary for any future health crises. Therefore, this research proposes an approach capable of targeting the hypotheses and eliminating the anomalies of Google Trends, thus reducing the likelihood of running into spurious correlations and having statistically uncertain outcomes. Specifically, the ability to predict the COVID-19 vaccination trend in Italy based on vaccine-related web queries is examined.

## Methods

### Procedure Summary

The hypothesis to be verified is that the COVID-19 “vaccine reservation” query (VRQ) can predict the trends of national and regional vaccinations (V). To achieve this scope and quantify the impact of mass media on web queries, cross-correlations between VQR, V, and COVID-19 vaccine–related headlines (VRH) of the Italian newspaper “La Repubblica” were searched. In particular, “La Repubblica” was chosen for its large readership and its online historical database (which allows the user to easily search for published articles containing a list of specific keywords). Besides, an appropriate regression model *V=f(VRH, VRQ)* was also constructed.

### Data Collection

The keyword “prenotazione vaccino” (vaccine reservation) was selected since it clearly expresses the desire to administer the dose of a vaccine. Synonyms of the word “prenotazione” (reservation) have been searched on the Treccani.it online dictionary. However, the synonym queries had a much lower relative search volume (RSV). Besides, even adding them to the original keyword through the “+” operator, the trends remained highly similar. Since the combination of queries makes it more likely that anomalies will appear in the data sets, a single query was chosen. The goodness of VRQ in identifying the web interest in COVID-19 vaccine queries is reported in the Results section. The Google Trends parameters have been set as follows: region: Italy; period: November 1, 2020, to November 27, 2021; category: all categories; and search type: web search. The “period” parameter has been changed to “Past 5 years” when performing a historical time series analysis. The “region” parameter was changed from “Italy” to “[the name of the region concerned]” when analyzing regional trends. The “interest over time” data sets were downloaded in “.csv format.” Following the previous methods, the keywords “disdire vaccino + cancellare vaccino + evitare vaccino + non vaccinarsi + green pass falso + comprare green pass” (revoke vaccine + cancel vaccine + avoid vaccine + do not get vaccinated + fake green pass + buy green pass) were searched to investigate users’ web interest in methods of not getting vaccinated. The first keyword searched was “disdire vaccino.” The other terms have been selected by consulting various possible synonyms in the Treccani.it online dictionary and Google Trends–related queries. The final exact queries searched on Google Trends are reported as references [17,18]. Regarding national vaccinations, the data set was downloaded from the “GitHub” platform [19]. The keyword “vaccino, vaccini, astrazeneca, pfizer, moderna, johnson&johnson, vaxzevria, comirnaty, pikevax” was searched in the historical archive of the newspaper “La Repubblica” [20]. In particular, this query includes the generic and proper names of the COVID-19 vaccines administered in Italy during the investigated period. The number of articles containing the aforementioned keyword was counted from week to week until it covered the period November 2020 to November 2021. The filter has been set to “ricerca avanzata” (advanced search) and “almeno una [parola]” (at least one [word]). This newspaper was chosen since it represents the second most widely read newspaper in Italy and provides the most detailed news database online. Furthermore, a previous publication showed similar news trends across primary Italian mass media during COVID-19 [21]. Such a result aligns with the theory of news competition and increasing returns-to-scale, which prompts profit-motivated media to publish on hot topics (as of interest to a broad audience) [22]. For these reasons, the author of this paper considered the source “La Repubblica” sufficient to represent the Italian media clamor about vaccines.

### Ethical Considerations

This study does not involve human participants or animals. All Google Trends data is anonymized. Therefore, the research does not require approval from a committee.

### Statistical Analysis

The shape of the data distribution was assessed both graphically and through the Shapiro-Wilk test. Since the data sets were not normal (*P*<.001) and above or below threshold correlations were not of interest, we adopted the Spearman correlation (*R*) [23]. To check the discrepancy between two time series, quantifiers such as percentage difference (used to compare the average RSV of two simultaneous series and indicated with “δ”) and percentage increase (used to compare the average RSV of two consecutive series and indicated with “Δ”) were exploited. The statistical significance of the discrepancies between average values was measured through the Welch *t* test (*t*), which is also valid for large nonnormal data sets [24,25]. When two contiguous time series were compared, a graphic check was carried out to guarantee the absence of seasonality and trends. All data sets were normalized to 100 by multiplying individual values by the constant “100/data set maximum value.” The “Lag week” was defined as the number of weeks by which a time series was shifted to obtain the maximum correlation with another time series. By doing so, it was possible to estimate the predictive power of one time series over another and the latency between them. Finally, a multiple regression was used to build the function Y=f(VRH, VRQ) to evaluate the impact of VRH and VRQ on V [26]. SEs for the regression coefficients are reported. Based on previous literature, any causal correlations between the media clamor and web searches should be sought within a maximum lag range of 3 weeks (from –3 to 3) [9,11,21,27,28]. Indeed, the web interest in a topic must arise around the media hype peak to be considered a direct consequence or cause of the latter. Regarding the pairs (VRH, V) and (VRQ, V), the lag acceptability range was fixed at 0 to 8 weeks since it can take up to 2 months from vaccine booking to administration. Fisher r-to-z transformation (*z*) was used to compare Spearman coefficients. Since the search for cross-correlations is highly exploratory, the Holm-Bonferroni correction was adopted (*m=50* hypotheses). The original *P* values have been reported alongside the adjusted ones (P*)—when P*>.001—to allow the reader to interpret the data independently.

### Mass Media Clamor as a Confounding Factor

As previously discussed, there is solid evidence that mass media can substantially impact users’ web interests. This fact increases the probability of spurious correlations due to a so-called confounding factor, defined as a “hidden” variable (or set of variables) capable of distorting the true relationship between other apparently correlated (or uncorrelated) variables [29]. In this specific case, media hype can create highly confounding scenarios. For example, a COVID-19 outbreak can generate intense news fanfare, immediately followed by a user’s growing web interest in the disease. After 7 days, an increase in COVID-19 cases is registered. Examining the sole couple (user interest, COVID-19 cases), it could seem like the online searches predicted the increase in infections. However, by introducing the “media hype” variable, it is observed that users’ web interest is much more correlated with the latter than with COVID-19 cases [21]. For this reason, media coverage is introduced in this analysis as a possible confounding factor capable of distorting the relationship between V and VRQ. In this regard, it is fair to admit that other confounding factors not considered in this paper could alter such a relationship in complex ways. Nonetheless, at present, to the best of the author’s knowledge, media influence is the only widely reported confounding factor in the literature regarding Google Trends. Furthermore, the main research hypothesis is well-targeted, thus reducing the likelihood of spurious correlations.

## Results

The adoption of the “vaccine reservation” query (VRQ) for our purpose is validated by the very strong correlation with the “covid vaccine” and “vaccine” queries (Multimedia Appendix 1, Figure S1) and the marked increase of its RSV in the period November 2020 to November 2021 compared to the past 4 years (Δ=11,500%; t_56_=6.8; P*<.001). The keywords related to the desire not to get vaccinated registered an average RSV of 4% compared to “vaccine reservation.” VRQ’s RSV has significantly exceeded that of searches for specific names such as “pfizer reservation,” “astrazeneca reservation,” “moderna reservation,” and “johnson&johnson reservation” (δ=190%; t_55_=6.6; P*<.001). Table 1 shows very strong correlations between VRQ and the national vaccination (V) trends (min *r*²=0.460; P*<.001, lag 0 weeks; max *r*²=0.903; P*<.001; lag 6 weeks). Significant correlations were also highlighted between VRQ’s RSV and the VRH of the newspaper “La Repubblica” (Multimedia Appendix 1, Table S1) and between VRH and V (Multimedia Appendix 1, Table S2). However, in these cases, the explained variations were markedly lower (max acceptable *r*^2^=0.237, *P*<.001, P*=.005, lag –3 weeks; max acceptable *r*^2^=0.286, *P*<.001, P*=.002, lag 8 weeks). The differences between the Spearman coefficients were highly significant (*z*=6.16, P*<.001; *z*=5.86, P*<.001).

**Table 1:** Spearman cross-correlations (R) between the “vaccine reservation” query (VRQ) and vaccination administrations in Italy from November 2020 and November 2021. The highest correlation is obtained by shifting the VRQ 6 weeks ahead.

Lag week	*R* (VRQ vs V^a^; 95% CI)	*P* value	P* value	N
–1	0.536 (0.297-0.711)	<.001	.002	47
0	0.678 (0.481-0.803)	<.001	<.001	48
1	0.777 (0.633-0.869)	<.001	<.001	48
2	0.833 (0.720-0.903)	<.001	<.001	48
3	0.887 (0.806-0.935)	<.001	<.001	48
4	0.927 (0.874-0.958)	<.001	<.001	48
5	0.946 (0.906-0.969)	<.001	<.001	48
6^b^	0.950 (0.912-0.971)	<.001	<.001	48
7	0.946 (0.905-0.969)	<.001	<.001	48

^a^V: vaccinations.

^b^The highest correlation is obtained by shifting the VRQ 6 weeks ahead.

**Figure 1:**
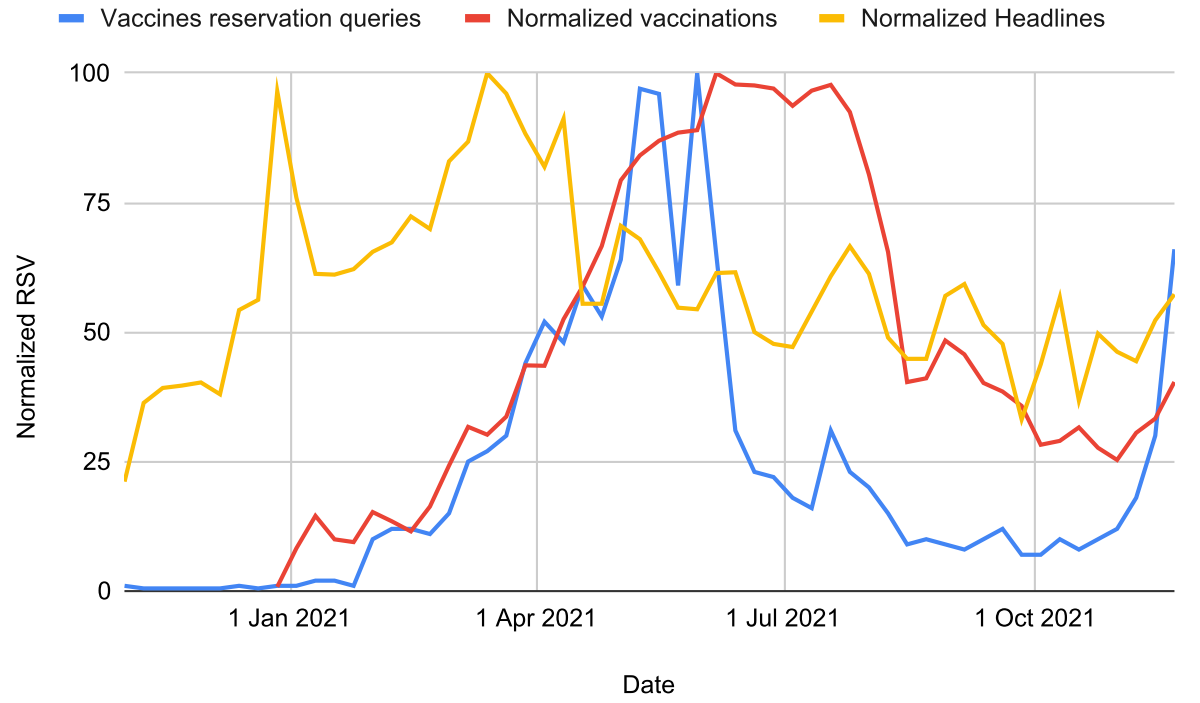
Comparison between the vaccine-related headlines of the newspaper "La Repubblica," national vaccinations, and national queries on vaccination reservations from November 2020 to November 2021. RSV: relative search volume.

The comparison of the trends is shown in Figure 1. All regional RSV trends have been similar to the national one (Multimedia Appendix 1, Figure S2) and were compatible with vaccination trends at the regional level [30]. Finally, the following regression model was built using appropriately translated time series based on the optimum lag previously identified (only values inside the acceptability range were considered): *Sqrt(V) = A + B × Log(VRH) + C × Log(VRQ)*, with *A*=–0.988 (SE 1.930; *P*=.61, P*>.99), *B*=2.67 (SE 1.16; *P*=.03, P*=.29), *C*=2.84 (SE 0.22; P*<.001). We observe that VRQ significance was greater than VRH. The following assumptions were considered verified: residual normality (Shapiro-Wilk *P*=.38), homoscedasticity (White test *P*=.77), and no multicollinearity (variance inflation factor [VIF]=1.46). Even considering an unlikely causal lag range of ±12 weeks, VRQ is the most significant variable to predict vaccinations: *Log(V) = A + B × Log(VRH) + C × Log(VRQ)*, with *A*=0.381 (SE 0.285; *P*=.19, P*>.99), *B*=0.487 (SE 0.180; *P*=.01, P*=.12), and *C*=0.353 (SE 0.041; P*<.001). Furthermore, despite that B>C, the 95% CIs are largely overlapping (overlap 0.308). The following assumptions were considered verified: residual normality (Shapiro-Wilk *P*=.86), homoscedasticity (White test *P*=.23), and no multicollinearity (VIF=2.45).

## Discussion

### Principal Findings

This study shows a marked and significant cross-correlation between web queries on vaccine reservations and actual vaccinations against COVID-19 in Italy. Based on the lower cross-correlations between vaccine-related news and vaccine web searches, the mass media may have only partially influenced web searches related to vaccine booking. Nevertheless, even assuming a positive impact of the mass media on these queries, this does not compromise the adoption of Google Trends as a predictive tool for vaccinations: indeed, the mass media could push users to search for online information on vaccines and then book their administration. Furthermore, COVID-19 vaccine reservation is easily obtainable through a user-friendly online procedure proposed by the regional health organizations (eg, [31]). This fact helps explain the strong correlation between web searches and vaccinations. Therefore, it is likely that the cross-correlations found between vaccine-related queries and vaccinations are not spurious. Alongside this, it is necessary to consider that the Italian mass media have even risked compromising the effectiveness of the vaccination campaign against COVID-19 by providing infodemic news on rare side effects [32]. Hence, it is plausible that, given the high number of vaccinations achieved at the national level, more authoritative sources have also been consulted by users. The capacity to provide accurate predictions on vaccination trends several weeks in advance is an extremely relevant epidemiological tool for developing future containment strategies [33]. These findings show that Google Trends can be exploited for this purpose if used properly. The search for simple well-targeted keywords on Google Trends is more likely to return the actual scenario of web interest on a certain topic. Specifically, it is essential not to use too complex or specific names, which tend to be ignored by users, and to try to express a precise action (in this case, the vaccine reservation).

Among the limitations of this paper, it is fair to emphasize that no definitive causal evidence has been provided, and unknown confounders may have skewed the results in unpredictable ways. Moreover, the variability of time lags between online booking and vaccine administration was not considered in this study. Finally, although well targeted, there are no guarantees that all the keywords relating to the desire not to be vaccinated have been selected. In this regard, given the broad antivaccination movement, many users may not have expressed an online interest in not getting vaccinated.

### Conclusions

This research provides preliminary evidence in favor of using Google Trends as a surveillance and prediction tool for vaccine adherence against COVID-19 in Italy. Further research is needed to establish appropriate use and limits of Google Trends for vaccination tracking. However, these findings prove that the search for suitable keywords is a fundamental step to reduce confounding factors. Additionally, targeting hypotheses helps diminish the likelihood of spurious correlations. It is recommended that Google Trends be leveraged as a complementary infoveillance tool by government agencies to monitor and predict vaccine adherence in this and future crises by following the methods proposed in this manuscript.

## Appendix

Multimedia Appendix 1 Supplementary figures and tables.

## Abbreviations

RSV: relative search volume
VIF: variance inflation factor
VRH: vaccine-related headlines
VRQ: vaccine reservation query
