# Supplementary material for: Google Trends as a Predictive Tool for COVID-19 Vaccinations in Italy: Retrospective Infodemiological Analysis
Source: JMIRx Med. 2022 Apr 19;3(2):e35356. doi: 10.2196/35356 (PMC9031689; doi:10.2196/35356)
Supplement: Multimedia Appendix 1 [file xmed_v3i2e35356_app1.docx]

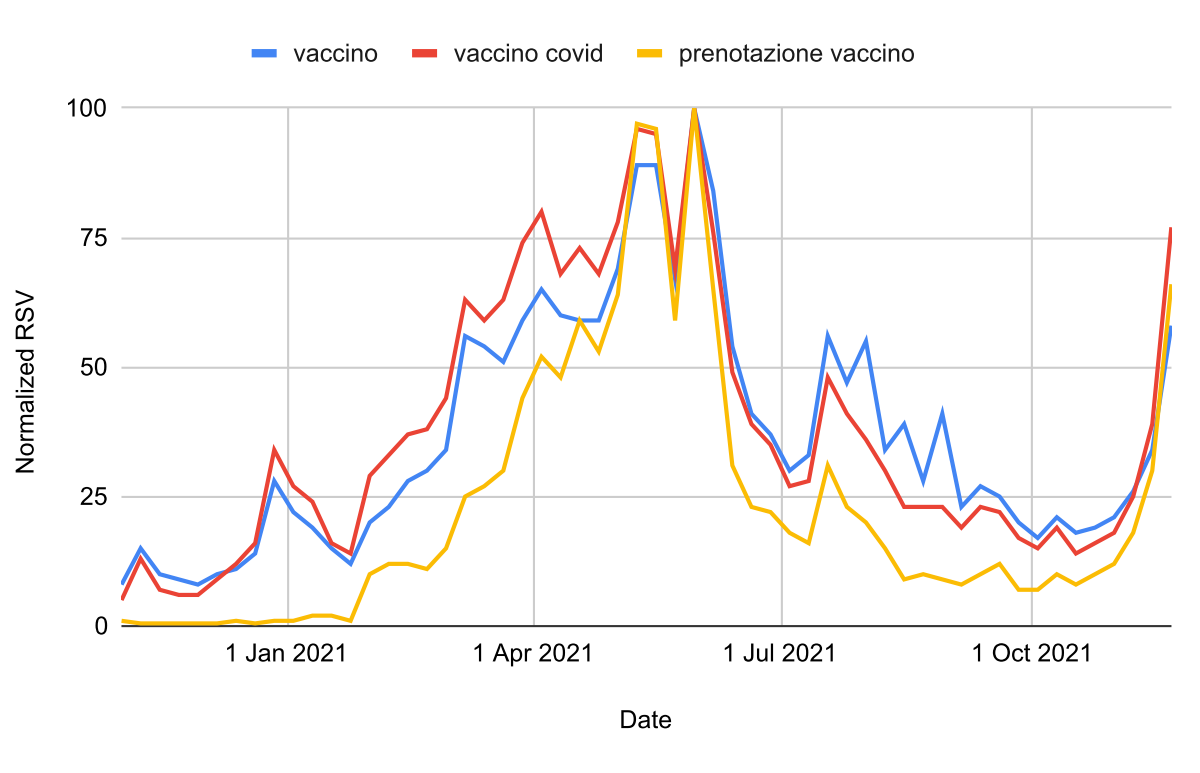
**Figure S1.** Normalized relative search volumes of the queries: "vaccino" (vaccine), "vaccino covid" (covid vaccine), and "prenotazione vaccino" (vaccine reservation).


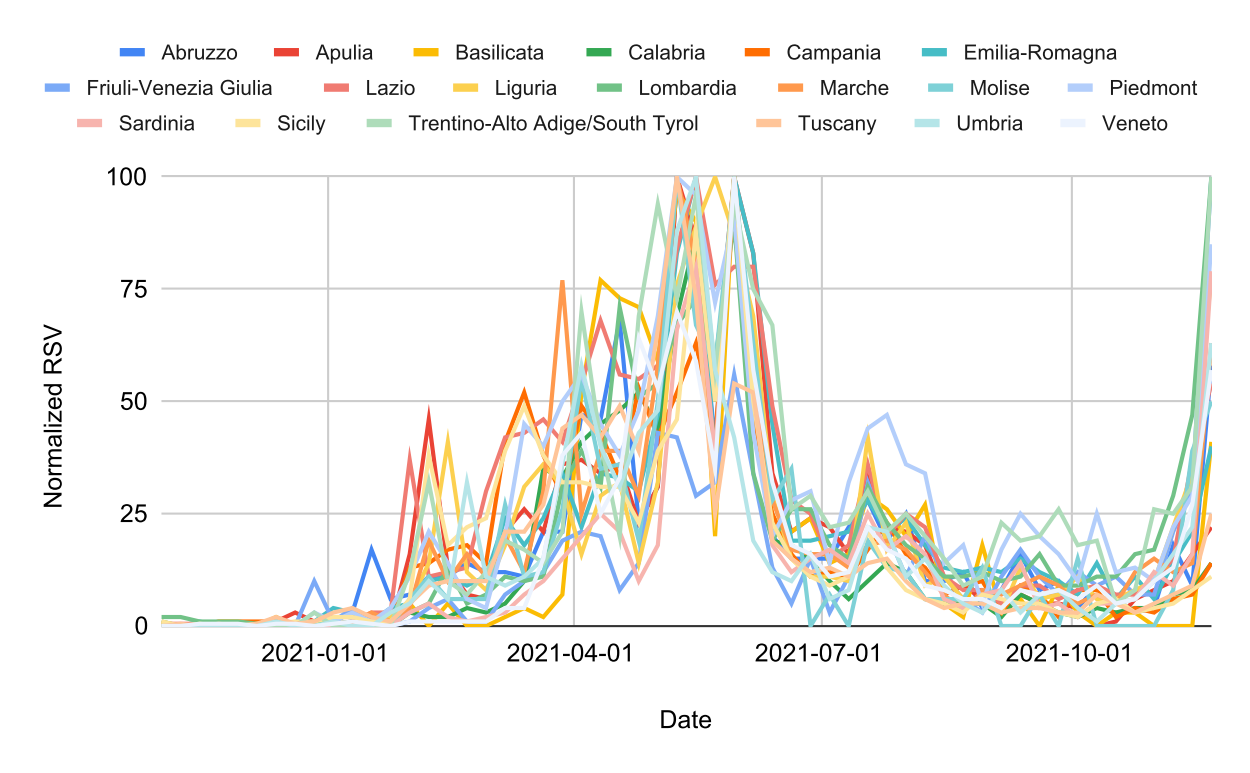
**Figure S2.** Comparison between the "prenotazione vaccino" (vaccine reservation) queries of all Italian regions from November 2020 to November 2021.

| Lag week | R (VRH vs VRQ) | 95% CI low | 95% CI up | P | P* | N |
| --- | --- | --- | --- | --- | --- | --- |
| -11 | 0.686 | 0.492 | 0.815 | <.001 | <.001 | 45 |
| -10 | **0.702** | **0.518** | **0.824** | **<.001** | **<.001** | **46** |
| -9 | 0.700 | 0.518 | 0.822 | <.000 | <.001 | 47 |
| -8 | 0.673 | 0.482 | 0.803 | <.001 | <.001 | 48 |
| -7 | 0.660 | 0.466 | 0.793 | <.001 | <.001 | 49 |
| -6 | 0.657 | 0.464 | 0.790 | <.001 | <.001 | 50 |
| -5 | 0.580 | 0.363 | 0.737 | <.001 | <.001 | 51 |
| -4 | 0.517 | 0.285 | 0.692 | <.001 | .002 | 52 |
| -3 *** | 0.487 | 0.250 | 0.668 | <.001 | .004 | 53 |
| -2 | 0.464 | 0.224 | 0.650 | .001 | .008 | 54 |
| -1 | 0.452 | 0.213 | 0.640 | .001 | .010 | 55 |
| 0 | 0.436 | 0.196 | 0.627 | .001 | .014 | 56 |

**Table S1.** Cross-correlations between the "vaccine reservation" query (VRQ) and “La Repubblica” vaccines-related headlines (VRH) from November 2020 and November 2021. The highest correlation is obtained by shifting VRQ ten weeks back. *** End of the a priori acceptability range.

| Lag week | R (VRH vs V) | 95% CI low | 95% CI up | P | P* | N |
| --- | --- | --- | --- | --- | --- | --- |
| -5 | -0.461 | -0.669 | -0.187 | .002 | .028 | 43 |
| -4 | -0.476 | -0.677 | -0.209 | .001 | .017 | 44 |
| -3 | -0.425 | -0.639 | -0.150 | .004 | .051 | 45 |
| -2 | -0.313 | -0.553 | -0.025 | .034 | .342 | 46 |
| -1 | -0.241 | -0.494 | 0.050 | .133 | .822 | 47 |
| 0 *** | -0.194 | -0.450 | 0.095 | .186 | >.999 | 48 |
| 1 | -0.119 | -0.390 | 0.171 | .420 | >.999 | 48 |
| 2 | -0.076 | -0.353 | 0.213 | .607 | >.999 | 48 |
| 3 | 0.020 | -0.266 | 0.302 | .895 | >.999 | 48 |
| 4 | 0.161 | -0.129 | 0.426 | .273 | >.999 | 48 |
| 5 | 0.295 | 0.012 | 0.534 | .042 | .376 | 48 |
| 6 | 0.403 | 0.134 | 0.617 | .005 | .059 | 48 |
| 7 | 0.458 | 0.200 | 0.657 | .001 | .018 | 48 |
| 8 *** | 0.535 | 0.296 | 0.711 | <.001 | .002 | 48 |
| 9 | 0.600 | 0.378 | 0.757 | <.001 | <.001 | 47 |
| 10 | 0.666 | 0.466 | 0.801 | <.001 | <.001 | 46 |
| 11 | 0.729 | 0.554 | 0.842 | <.001 | <.001 | 45 |
| 12 | **0.781** | **0.630** | **0.875** | **<.001** | **<.001** | **44** |
| 13 | 0.773 | 0.616 | 0.871 | <.001 | <.001 | 43 |

**Table S2.** Cross-correlations between “La Repubblica” vaccines-related headlines (VRH) and vaccination administrations in Italy from November 2020 and November 2021. The highest correlation is obtained by shifting the VDH twelve weeks ahead. *** Edge of the a priori acceptability range.
